# The molecular interplay among gut dysbiosis, adipose tissue, and metabolite-derived damage-associated molecular patterns in metainflammation and atherogenesis

**DOI:** 10.3389/fimmu.2025.1694061

**Published:** 2025-11-26

**Authors:** Leslie Marisol González-Hermosillo, Karol Iliana Ávila-Soto, Lucía Angélica Méndez-García, Arturo Cérbulo-Vázquez, Marcela Esquivel-Velázquez, Nallely Bueno-Hernández, Miguel Ángel Fonseca-Sánchez, Galileo Escobedo

**Affiliations:** 1 Laboratorio de Immunometabolismo, Dirección de Investigación, Hospital General de México “Dr. Eduardo Liceaga”, Mexico City, Mexico; 2 Laboratorio de Proteómica, Dirección de Investigación, Hospital General de México “Dr. Eduardo Liceaga”, Mexico City, Mexico; 3 Dirección de Investigación, Hospital General de México “Dr. Eduardo Liceaga”, Mexico City, Mexico

**Keywords:** metainflammation, low-grade systemic inflammation, monocyte-derived macrophages, metabolite-derived damage-associated molecular patterns, low-density lipoproteins, glucose, short-chain fatty acids, gut microbiome

## Abstract

Metainflammation is a low-grade systemic inflammatory response that can persist for months or even years, during which monocytes, macrophages, and other immune cells become hyperactivated, contributing to metabolic disease and atherogenesis. Although we now better understand the role of metainflammation in atherosclerosis, uncertainty persists about how gut dysbiosis, adipose tissue expansion, and metabolite-derived damage-associated molecular patterns (Md-DAMPs) can trigger metainflammation and promote atherogenesis. In this comprehensive review, we summarize the role of gut dysbiosis in lipopolysaccharide (LPS) production, a component of gram-negative bacteria that can trigger metainflammation by stimulating circulating monocytes and tissue-resident macrophages. We also outline adipose tissue expansion as an additional igniter of metainflammation by driving the expression of hypoxia-inducible factor 1α (HIF-1α), a master transcription factor that leads to nuclear factor kappa B (NFκB)-dependent proinflammatory cytokine production. Furthermore, we thoroughly explored the precise nature of Md-DAMPs, including glutamate, bile acids, lipoproteins, short-chain fatty acids (SCFAs), uric acid, and excess glucose, with emphasis on the molecular mechanisms that mediate their roles in metainflammation and atherosclerosis. Finally, we integrate the molecular interplay among gut dysbiosis, adipose tissue expansion, and Md-DAMPs to a scenario in which circulating monocytes, macrophages, and foam cells contribute to atherosclerotic plaque formation, instability, and rupture. In conclusion, the information examined here may help refresh our conceptual understanding of atherogenesis, incorporating novel actors as gut dysbiosis, adipose tissue expansion, and Md-DAMPs in the complex network that leads to metainflammation and cardiovascular disease.

## Introduction

A growing body of evidence in animal models and patients with cardiovascular risk has shown a central connection between systemic inflammation and atherosclerosis development, leading to abdominal aortic aneurysms, stroke, or coronary artery disease (1–4). Systemic inflammation has distinctive features that differ from classic inflammation, especially in three crucial attributes. First, systemic inflammation is characterized by abnormally high circulating levels of acute-phase proteins and proinflammatory cytokines such as C-reactive protein (CRP), tumor necrosis factor-alpha (TNF-alpha), interleukin (IL-) 1 beta, IL-6, and IL-12, among others (5–7). However, these proinflammatory mediators do not increase to the same extent as in inflammatory conditions such as sepsis, ulcerative colitis, or rheumatoid arthritis (8–10). Second, systemic inflammation promotes the recruitment of monocytes and other immune cells into peripheral tissues, including skeletal muscle, visceral and subcutaneous fat, and blood vessels (11–13). Monocytes are myeloid lineage cells with enormous plasticity that can adopt either tissue patrolling functions as classical or intermediate monocytes or inflammatory activity as non-classical monocytes, depending on the immune and non-immune microenvironment (14, 15). After infiltrating a tissue, monocyte subtypes can differentiate into macrophages, which begin to produce proinflammatory mediators and chemokines that attract other immune cells, thus perpetuating the local inflammatory environment (16, 17). Monocyte-derived macrophages (MDMs) do not directly cause tissue damage or necrotic lesions, suggesting that immune cell hyperactivity in systemic inflammation is low-grade (18, 19). Third, low-grade systemic inflammation is a long-term immune response where circulating cytokine levels and MDM hyperactivity can persist for months, even years, contributing to the onset of metabolic alterations and cardiovascular disease (20, 21). These notions underlie many global research teams that have coined the term “metainflammation” to describe a state of immune hyperactivity driven by metabolic dysfunction.

Despite understanding the relationship among metainflammation, metabolic disturbances, and cardiovascular disease, numerous questions remain. Who is behind unleashing immune hyperactivation of monocyte subsets and MDMs? In other words, as Prof. Dr. Jaap G. Neels and Prof. Dr. Jerrold M. Olefsky masterfully questioned almost twenty years ago, what starts the fire? (22). In this regard, this comprehensive review will address the primary molecular mechanisms behind gut dysbiosis-derived lipopolysaccharide (LPS) and adipose tissue expansion in immune cell activation and migration, cytokine production, and metainflammation. Another inquiry still elusive is how metabolite-derived damage-associated molecular patterns (Md-DAMPs), including glutamate, bile acids, short-chain fatty acids (SCFAs), oxidized low-density lipoproteins (OxLDLs), uric acid, or excess glucose, contribute to monocyte phenotype, MDM activation, and metainflammation? Finally, this thorough review will integrate the synergistic interactions among Md-DAMPs, monocyte subsets, macrophages, and foam cells in the development of endothelial dysfunction and injury, atheroma formation, and atherosclerotic plaque instability and rupture in the context of metainflammation.

## What starts the fire?

**Figure 1:**
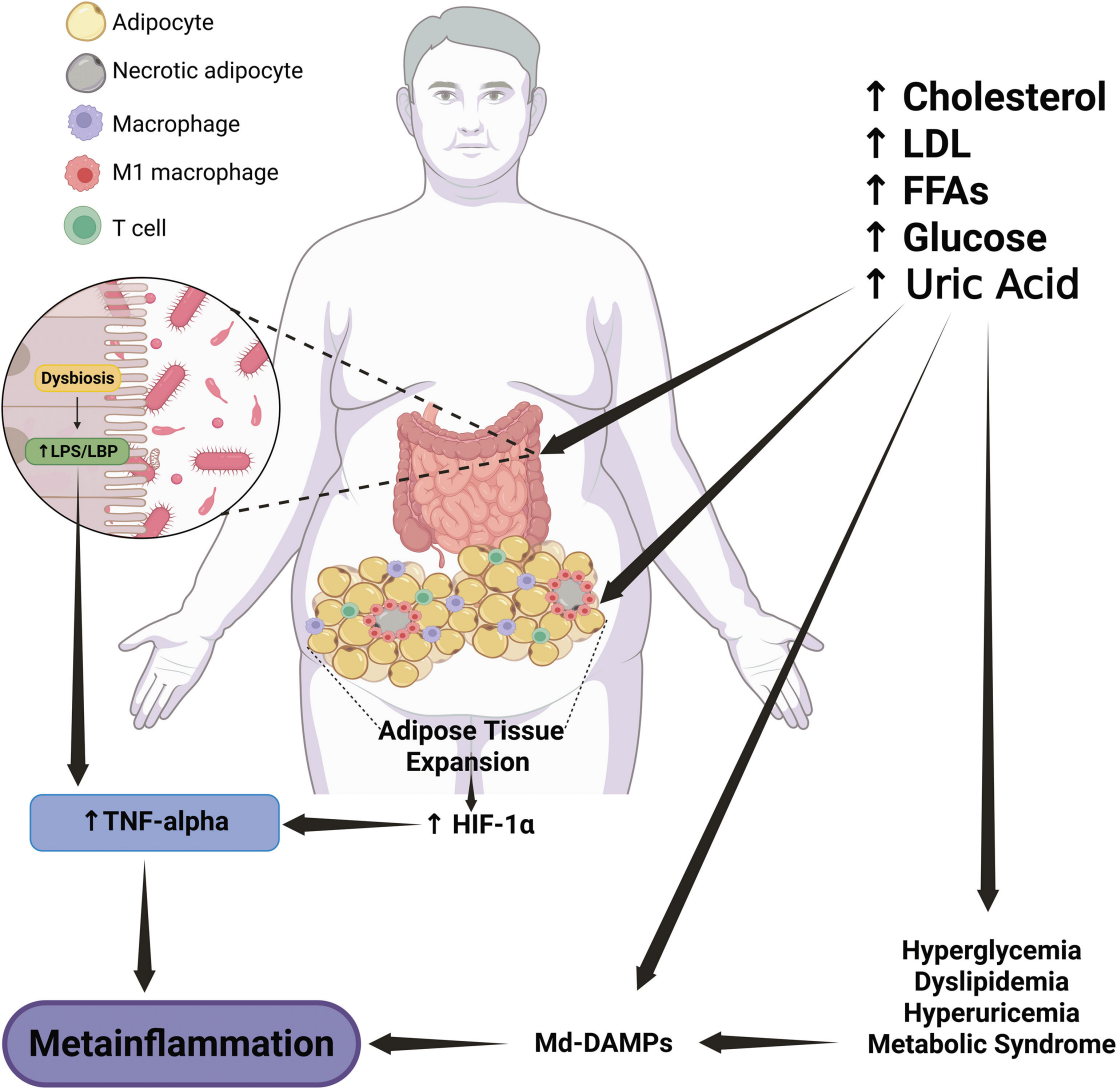
Role of Md-DAMPs in atherosclerotic plaque progression. Pathological entities related to metabolic syndrome, such as hyperglycemia, dyslipidemia, and hyperuricemia, are the primary sources of Md-DAMPs, including LDL, oxLDL, uric acid, and excess glucose. In parallel, metabolic syndrome-related alterations also induce endothelial damage and dysfunction, leading to endothelial cell death, oxidative stress, and immune cell recruitment, particularly circulating monocytes that can infiltrate the tunica intima. In an environment enriched in Md-DAMPs, monocytes tend to adopt a proinflammatory profile similar to that of non-classical monocyte subsets, with increased IL-1 beta production and enhanced vascular patrolling and trafficking. Recruited monocytes differentiate into M1 proinflammatory macrophages, which engulf lipoproteins and produce TNF-alpha, thus turning macrophages into foam cells and starting the process of atherosclerotic plaque formation. Circulating monocytes continue to infiltrate and differentiate into M1 macrophages, which will turn into foam cells and produce proinflammatory cytokines, MCP-1, and EMPs. This milieu, enriched in Md-DAMPs, inflammatory mediators, and profibrotic signals, promotes immune cell recruitment and fibrous cap formation, thus leading to plaque growth and stability in a first stage. In a late phase, TNF-alpha also induces apoptosis of smooth muscle cells and endothelial cells within the vascular endothelium. At the same time, IL-1 beta stimulates the expression of MMPs with the ability to degrade collagen and other MEPs. This scenario results in thinning and weakening of the fibrous core, which promotes plaque instability and rupture and leads to coronary artery disease and myocardial compromise. An upward arrow indicates an increase. A downward arrow indicates a decrease. Connector arrows indicate a causative relationship, with the arrow pointing from the cause to the effect. Abbreviations: Md-DAMPs, metabolite-derived damage-associated molecular patterns; TA, tunica adventitia; TM, tunica media; TI, tunica intima; LDL, low-density lipoprotein; oxLDL, oxidized low-density lipoprotein; UA, uric acid; Glu, glucose; TNF-alpha, tumor necrosis factor alpha; IL, interleukin; MCP-1, monocyte chemoattractant protein-1; EMPs, extracellular matrix proteins; MMPs, matrix metalloproteinases; SMCs, smooth muscle cells; CAD, coronary artery disease.

The primary triggers of metainflammation are still a hot topic in immunometabolism and physiology. A solid body of evidence indicates that intestinal dysbiosis and adipose tissue expansion are the leading drivers of immune hyperactivation (**Figure 1**), particularly in monocyte subsets and macrophages (23–26).

For decades, we all believed that intestinal flora was just gut bacteria we should keep under control with an enormous variety of antibiotics, often misused without medical supervision and prescription-free (27, 28). Nevertheless, the gut microbiome is a 2-kilogram organ composed of more than 400 bacterial species that are responsible for carbohydrate breakdown, SCFA synthesis, vitamin production, immune system maturation, protection against harmful bacterial colonization, and maintenance of intestinal barrier integrity, among other functions (29–35). Bacteria within the human intestine are in a delicate but complex balance. Intestinal dysbiosis occurs after disruption of intestinal flora equilibrium, altering bacterial composition and function and allowing the proliferation of detrimental microorganism species, such as gram-negative bacteria (36, 37). Gram-negative bacteria include more than 100 species of microorganisms responsible for most in-hospital morbidity and mortality, as well as antibiotic resistance. Bacterial species such as *Chlamydia* sp., *Escherichia coli*, *Klebsiella* sp., *Pseudescherichia* sp., *Salmonella* sp., *Shigella* sp., *Neisseria* sp., and *Helicobacter pylori* are perhaps the most well-known gram-negative bacteria responsible for lipopolysaccharide (LPS) production (38, 39). LPS is a complex molecule composed of repeated oligosaccharide units, core sugars, and lipid A, and it is a crucial outer membrane component of bacteria (40). Dysbiosis results in LPS release into the gut lumen, where this endotoxin can bind to lipopolysaccharide-binding protein (LBP), a soluble acute-phase protein primarily produced in the liver and intestine (41). CD14 on monocytes and macrophages can bind the LPS-LBP complex, inducing dimerization of toll-like receptor 4 (TLR4)/myeloid differentiation factor 2 (MD2) and activating myeloid differentiation primary response 88 (MyD88) (42). The TLR4/MD2-MyD88 signaling pathway initiates the canonical cascade of proinflammatory events, leading to activation of IκB kinase (IKK), which, in turn, phosphorylates IκBα, resulting in nuclear factor kappa B (NFκB) dimerization (43). The NFκB dimer can translocate to the nucleus, where it acts as a transcription factor, inducing the expression of cytokines and chemokines such as TNF-alpha, pro-IL-1 beta, IL-6, IL-12, and monocyte chemoattractant protein-1 (MCP-1) (44). This evidence may partially explain how intestinal dysbiosis leads to elevated blood LPS levels, and it also provides a molecular basis for understanding why monocytes and macrophages show an increased ability to produce inflammatory mediators systemically. In this regard, circulating monocytes exhibit a higher capacity to produce TNF-alpha in obesity and type 2 diabetes (T2D), both conditions characterized by elevated LPS and LBP serum levels (45–47). Notably, Sakura et al. reported that men with T2D also showed a significant correlation between serum LBP and aortic pulse wave velocity, a widely used measurement of arterial stiffness that serves as a surrogate marker of subclinical atherosclerosis (47). A study in mice with high-fat diet (HFD)-induced insulin resistance found that increased TNF-alpha levels promote a rise in circulating monocytes, thereby perpetuating and amplifying metainflammation (48). This information concurs with results from mouse models showing that antibiotic-induced intestinal dysbiosis is related to increased IL-1 beta-producing CD68+ macrophages in aortic atherosclerotic plaques, accompanied by higher LPS serum levels (49, 50). Altogether, this evidence supports the role of intestinal dysbiosis as a primary source of systemic LPS, which is an endotoxin that can trigger immune hyperactivation in monocytes and macrophages by promoting proinflammatory cytokine release into the bloodstream.

Adipose tissue expansion refers to the enlargement in size or number of adipose cells within the fat mass, also referred to as hypertrophy and hyperplasia, respectively (51). Adipose tissue expansion is a histologic feature of obesity, primarily occurring in visceral and subcutaneous fat depots around internal organs in the abdominal cavity or the hypodermis in areas such as the abdomen, thighs, and buttocks (51, 52). Adipose tissue hyperplasia is a common feature of fat mass in children and adolescents with weight gain (53). Conversely, adipose tissue hypertrophy is a hallmark of obesity in adulthood, as well as long-term insulin resistance and metabolic disturbances (54). Accumulating evidence in animal models and humans suggests that adipose tissue expansion may also play a crucial role in metainflammation development by starting a local inflammatory cascade that can expand to the rest of the body.

During obesity, fatty acids transported by chylomicrons increase in the bloodstream (55, 56). Then, lipoprotein lipase hydrolyzes chylomicrons in adipose tissue’s vascular stroma, thereby releasing free fatty acids that adipocytes can store as triglyceride droplets via re-esterification (57). Consequently, adipocytes become expanded cells. Adipocyte expansion is a human trait resulting from millions of years of evolution as hunter-gatherers in a new world where the first hominids had to run to catch food (58–60). In this scenario, it is unsurprising that excessive weight gain is associated with the widespread availability of high-sugar, high-fat foods, which have contributed to adipose tissue enlargement, along with other factors such as genetic background and lack of physical activity. Bigger adipose cells require more oxygen, which is available in the proximity of vascular stroma’s capillaries (61, 62). However, adipocytes located far from stromal capillaries may have reduced oxygen bioavailability, leading to hypoxia-inducible factor 1α (HIF-1α) production (63, 64). HIF-1α is a master transcription factor that orchestrates the expression of a wide range of genes involved in hypoxia adaptation, including glycolysis, erythropoiesis, vascular endothelial growth factor (VEGF)-mediated angiogenesis, cell survival, and even apoptosis (65–68). HIF-1α also acts as an immune function regulator by promoting NFκB-dependent TLR4 expression, TNF-alpha and IL-6 synthesis, phagocytosis, and macrophage migration mediated by MCP-1 (69–72). Under prolonged hypoxia, adipocytes produce HIF-1α, which induces downstream expression of MCP-1, a chemokine that attracts circulating monocytes that can infiltrate adipose tissue and differentiate into MDMs (73). The HIF-1α-induced microenvironment, enriched in TNF-alpha and IL-6, instigates infiltrated MDMs and adipose tissue macrophages (ATMs) to adopt a proinflammatory activation state and form crown-like structures characteristic of necrotic adipose tissue (74). In this way, adipose tissue expansion, hypoxia, MDMs, and ATMs work synergistically to unleash an orchestrated inflammatory cascade that initiates metainflammation and propagates a systemic milieu favorable to the development of atherosclerotic disease.

This information indicates that gut dysbiosis and adipose tissue expansion are central igniters of metainflammation through LPS production and HIF-1α activation, thereby simultaneously amplifying a proinflammatory environment favorable for atherosclerosis. Once initiated, metainflammation elicits the release of Md-DAMPs, which are metabolic signals that can directly stimulate immune cells to produce proinflammatory cytokines, chemokines, and reactive oxygen species (ROS) via specific receptors and intracellular pathways.

## Role of Md-DAMPs in metainflammation

Prof. Dr. Seung-Yong Seong and Prof. Dr. Polly Matzinger first proposed the term damage-associated molecular patterns (DAMPs) in 2004 to describe a variety of ligands released from injured or dying cells able to trigger innate immune responses, including sterile inflammation (75). Fourteen years later, our research team was among the first to coin the term Md-DAMP to describe endogenous metabolic signals that polarize immune cells toward a proinflammatory profile and contribute to the development of cardiovascular disease (76). Currently, a growing number of research groups have consistently reported various Md-DAMPs, including glutamate, bile acids, lipoproteins, SCFAs, uric acid, and excess glucose, among others (76–78). Compared to prototypical immune ligands such as LPS or TNF-alpha, Md-DAMPs are non-prototypical molecules with immunoregulatory actions in numerous innate immune cells, including monocyte subsets, MDMs, ATMs, eosinophils, neutrophils, and mast cells (78, 79). Unlike classical pathogen-associated molecular patterns (PAMPs) or DAMPs, Md-DAMPs are produced from altered metabolic processes rather than from pathogens or injured cells. In fact, the primary sources of Md-DAMPs are metabolic alterations such as hyperglycemia, insulin resistance, dyslipidemia, and hyperuricemia, among others, which increase the thresholds of molecules as glucose, lipoproteins, glutamate, and uric acid (80, 81). Finally, Md-DAMPs are etiopathogenic factors of metainflammation that lead to the development of critical entities such as T2D, metabolic dysfunction-associated steatotic liver disease (MASLD), chronic kidney disease, hypertension, and atherosclerosis (**Table 1**). Given the definition of Md-DAMP, we will now examine the molecular mechanisms by which Md-DAMPs influence immune cell activity in the context of metainflammation and cardiovascular disease, with particular emphasis on atherosclerosis.

**Table 1:** Summary of the effects of Md-DAMPs on immune cells involved in metainflammation and atherogenesis.

Md-DAMP	Primary triggers	Associated receptors or signaling pathways	Immune cell targets	Main effects	Role in metainflammation and atherogenesis	References
Glutamate	ATE→HIF-1α →Glutaminase	NMDAr, mGR1, mGR5, PKC, PLC, ERK, MAPK, NFκB	CD8+ T cells	↑ IFN-gamma	T2D pathogenesis, insulin resistance, and vascular damage	(77, 79, 83–96)
			Macrophages	↑ TNF-alpha		
			Mast cells	↑ IL-6		
			Neutrophils	↑ ROS		
Bile acids DCA, CDCA, TLCA	High-fat western diets→ATE→ HIF-1α	FXR, PXR, VDR, S1PR2, TGR5	Dendritic cells	↑ IL-1 alpha and IL-1 beta	Inflammatory systemic responses in obesity, T2D, MASLD, and atherosclerosis with dual anti-inflammatory actions depending on target receptor	(97–104)
			Monocytes	↑ Cell trafficking		
			Neutrophils	↑ Cell trafficking		
			MDMs	↓LPS-induced expression of IL-1 alpha, IL-6, and TNF-alpha		
Native LDL and ox-LDL	High-fat western diets→ATE→ HIF-1α	CD36, LOX-1, NFκB, NLRP3	Monocytes	↓ Classical subset ↑ Non-classical subset ↑ IL-1 beta	Foam cell formation, chronic vascular inflammation, and atherosclerotic plaque progression	(106–112)
			Macrophages	↑ TNF-alpha and IL-6 ↓ IL-10 ↑ ROS		
			MDMs	↑ IL-1 beta		
HDL deficiency	High-fat western diets→ATE→ HIF-1α	Caveolin-1, ERK, STAT3	Monocytes	↑ Non-classical subset ↑ IL-1 beta	Inflammatory activation of monocytes and macrophages with atherogenic functions	(113, 114)
			Macrophages	↑ M1 polarization ↑ IL-6, TNF-alpha, MCP-1, and ROS		
SCFAs acetate, propionate, butyrate	High-fat western diets→Gut dysbiosis→ LPS	GPR41, TLR4, NFκB	PBMCs	↑ IL-1 beta, IL-6, and IL-8 at high concentrations ↓ TNF-alpha and IL-6 at low concentrations	Gut dysbiosis leads to SCFAs imbalance, which triggers metainflammation, T2D, and atherogenesis	(117–122)
			BM-DM	↓ M1 polarization and IL-6 at high concentrations		
Uric acid	High-fat and carbohydrate western diets→ ATE→HIF-1α	URAT1, TLR2, TLR4, NOD-like receptors, NLRP3, NFκB	MDMs	↑ TNF-alpha, IL-1 alpha, and IL-1 beta ↑ ROS ↑ Phagocytic activity	Enhancement of M1 polarization and infiltration in atherosclerotic lesions. Promotion of plaque formation	(76, 133–137)
Glucose	High-carbohydrate western diets→ ATE→HIF-1α	TLR4, RAGE, GLUT1, HIF-1α, NFκB, epigenetic regulation	MDMs	↑ iNOS ↓ IL-10	Hyperglycemia-derived metainflammation, T2D, vascular inflammation, and plaque instability and rupture	(140–149)
			Monocytes	↑ IL-1 beta, IL-6, and TNF-alpha		
			Macrophages	↑ IL-1 beta, IL-6, TNF-alpha, and MCP-1 ↓ methylation in H3K9me3 leading to IL-6 production		

The first column shows the sort of Md-DAMP, followed by receptors or signaling pathways potentially involved in mediating the actions, immune cell target, main effects, primary role in atherogenesis, and references containing the source of information. Upwards arrow indicates increase. Downwards arrow indicates decrease. Abbreviations: Md-DAMPs, metabolite-derived damage-associated molecular patterns; ATE, adipose tissue expansion; HIF-1α, hypoxia-inducible factor 1 alpha; NMDAr, N-methyl-D-aspartate receptor; mGR1, metabotropic glutamate receptor 1; mGR5, metabotropic glutamate receptor 5; PKC, protein kinase C; PLC, phospholipase C; ERK, extracellular signal-regulated kinase; MAPK, mitogen-activated protein kinase; NFκB, nuclear factor kappa B; CD, cluster of differentiation; IFN-gamma, interferon gamma; TNF-alpha, tumor necrosis factor alpha; IL, interleukin; ROS, reactive oxygen species; T2D, type 2 diabetes; DCA, deoxycholic acid; CDCA, chenodeoxycholic acid; TLCA, taurolithocholic acid; FXR, farnesoid X receptor; PXR, pregnane X receptor; VDR, vitamin D receptor; S1PR2, sphingosine-1-phosphate receptor 2; TGR5, Takeda G-protein-coupled receptor 5; MDMs, monocyte-derived macrophages; LPS, lipopolysaccharide; MASLD, metabolic dysfunction-associated steatotic liver disease; LDL, low-density lipoprotein; ox-LDL, oxidized low-density lipoprotein; LOX-1, lectin-like oxidized LDL receptor-1; NLRP3, nucleotide-binding and oligomerization domain (NOD)-like receptors, and nucleotide-binding domain, leucine-rich-containing family, pyrin domain-containing 3; HDL, high-density lipoprotein; STAT3, signal transducer and activator of transcription 3; MCP-1, monocyte chemoattractant protein-1; SCFAs, short-chain fatty acids; GPR41, G-protein coupled receptor 41; TLR4, toll-like receptor 4; TLR2, toll-like receptor 2; PBMCs, peripheral blood mononuclear cells; BM-DM, bone marrow-derived macrophages; M1, proinflammatory macrophage activation; NOD, Nucleotide-binding oligomerization domain, leucine-rich repeat containing receptors; URAT1, urate transporter 1; RAGE, receptor for advanced glycation end-products; GLUT1, glucose transporter type 1; iNOS, inducible nitric oxide synthase.

Glutamate is a neurotransmitter produced by neurons and glial cells that exerts its actions through binding to ionotropic (iGR) or metabotropic (mGR) receptors (82). Notably, hypertrophic white adipose tissue from mice with diet-induced obesity is also a source of glutaminase-mediated glutamate production that concurs with reduced adiponectin secretion and decreased insulin-dependent glucose uptake (83, 84). Besides acting as an excitatory neurotransmitter in the central nervous system, glutamate can behave as an Md-DAMP and increase the production of interferon-gamma (IFN-gamma) in cytotoxic T cells and TNF-alpha in macrophages via mGR1 or mGR5 (79, 85). In CD8+ T cells, glutamate binds to mGR1 and activates phospholipase C (PLC), which in turn cleaves phosphatidylinositol 4,5-bisphosphate (PIP2) into inositol trisphosphate (IP3) and diacylglycerol (DAG) (86). IP3 and DAG are crucial second messengers that promote calcium (Ca^2+^) release from the endoplasmic reticulum and activate protein kinase C (PKC), respectively (86). The increase in intracellular Ca^2+^ and phosphorylated PKC activates prominent transcription factors involved in IFN-gamma expression, including nuclear factor of activated T cells (NFAT) and activator protein-1 (AP-1) (87, 88). In parallel, glutamate interaction with mGR5 leads to extracellular-signal-regulated kinase (ERK) phosphorylation and mitogen-activated protein kinase (MAPK) activation, which promote IFN-gamma expression, like that observed with T-cell receptor (TCR) stimulation (89). In macrophages, glutamate interaction with mGR5 activates the PLC-dependent PKC signaling pathway, which in turn promotes NFκB dimerization and translocation to the nucleus, where it upregulates TNF-alpha transcription (90, 91). Simultaneously, the IP3-dependent intracellular Ca^2+^ increase also triggers TNF-alpha production via calmodulin-dependent kinases (CaMK), thus contributing to glutamate proinflammatory effects on macrophages (92). The role of glutamate in immune cells also extends to polymorphonuclear leukocytes and iGRs, as in murine bone marrow-derived mast cells, which exhibit increased IL-6 synthesis via the N-methyl-D-aspartate receptor (NMDAr) (77). The use of MK-801, an NMDAr antagonist, demonstrated that iGRs mediate glutamate-dependent IL-6 production in mast cells (77). Primary human neutrophils exhibit a progressive, dose-dependent decrease in ROS production when cultured with Co101244, another NMDAr antagonist (93). This body of evidence aligns with the increased glutamate levels observed in patients with T2D, a complex metabolic disease characterized by abnormally high blood glucose levels that significantly increase the risk of atherosclerotic plaque formation (94). With apparent effects on CD8+ T cells, macrophages, mast cells, and neutrophils, glutamate acts as an Md-DAMP via mGRs and iGRs, such as mGR1, mGR5, and NMDAr, expressed in both adaptive and innate immune cells that also play a role in metainflammation (95, 96).

Another type of molecule showing a dual role in metabolism and inflammation is bile acids. Bile acids are produced in hepatocytes and stored in the gallbladder. After dietary fat intake, bile acids are secreted into the duodenum to enable the digestion and absorption of fats. In addition to their roles in lipid metabolism, emerging evidence suggests that bile acids are aberrantly altered in obesity, T2D, and MASLD and act as Md-DAMPs, orchestrating either anti-inflammatory or proinflammatory responses, depending on their chemical structure and target receptors. For instance, deoxycholic acid (DCA) and chenodeoxycholic acid (CDCA) promote the *in vitro* secretion of IL-1 alpha and IL-1 beta in murine bone marrow-derived dendritic cells, thereby facilitating the recruitment of monocytes and neutrophils to inflamed tissues (97). Both DCA and CDCA are bile acids that share a 24-carbon steroid structure, differing only in the location of the second hydroxyl group, and act primarily through the farnesoid X receptor (FXR), the pregnane X receptor (PXR), the vitamin D receptor (VDR), and sphingosine-1-phosphate receptor 2 (S1PR2) (98, 99). Conversely, a study reported that the secondary bile acid taurolithocholic acid (TLCA) suppresses LPS-induced proinflammatory cytokine expression in primary human MDMs, including IL-1 alpha, IL-6, and TNF-alpha (100). TLCA is a secondary bile acid whose structure substantially differs from DCA and CDCA, showing the amino acid taurine attached to the steroidal backbone through an amide bond (101). Furthermore, TLCA primarily exerts its actions through the Takeda G-protein-coupled receptor 5 (TGR5), a receptor expressed in cytotoxic T cells and macrophages that suppresses antiviral and inflammatory responses (102–104). As outlined, bile acids act as Md-DAMPs by affecting cytokine expression and immune cell activation. However, the effects of bile acids on immune cells are not uniform, as they depend on bile acid structure and target receptors, which may partially explain conflicting evidence regarding their capacity to induce proinflammatory or anti-inflammatory responses. We still need to conduct further research to clarify the role of bile acids in immune activity, including the type of bile acid, the receptor involved, and the concentration thresholds, to understand how these metabolites contribute to metainflammation.

OxLDLs were among the first molecules described as Md-DAMPs due to their role in macrophage differentiation into foam cells, a critical component of atheromatous plaque formation (105). OxLDLs result from the oxidation of native low-density lipoproteins (LDLs), a group of lipophilic proteins synthesized in the liver as very-low-density lipoproteins (VLDLs) initially (106). In the context of dyslipidemia and metabolic dysfunction, monocytes infiltrating the subendothelial space of blood vessels can differentiate into macrophages with proinflammatory activities, including the expression of TNF-alpha and the production of ROS (106, 107). Macrophages also exhibit the ability to uptake oxLDLs via scavenger receptors, such as the cluster of differentiation (CD) 36 and lectin-like oxidized low-density lipoprotein receptor-1 (LOX-1), internalizing oxLDL molecules to form lipid droplets inside the cell (108). The cytosolic accumulation of lipids causes macrophages to transform into foam cells, which not only contribute to the formation of the atheromatous plaque but also enhance inflammatory functions, including the activation of the inflammasome and NFκB pathways, leading to the release of IL-1 beta and TNF-alpha (109).

Nevertheless, accumulating evidence indicates that oxLDLs are not the only lipoproteins that act as Md-DAMPs in metainflammation, metabolic dysfunction, and atherosclerosis. Native LDL also appears to function as an Md-DAMP, affecting a variety of innate immune cells involved in atherogenesis. A previous study from our group demonstrated that native LDLs synergistically act with LPS to decrease CD14++CD16+ classical monocytes and increase CD14+CD16+ non-classical monocytes via CD36, both *in vitro* and in patients with increased atherogenic risk (110). Another report indicated that native LDL stimulates the differentiation of all monocyte subtypes into IL-6 and TNF-alpha-producing macrophages, which also exhibit a reduced ability to synthesize the anti-inflammatory cytokine IL-10 (111). Consistent with these data, native LDL induces inflammasome activation and IL-1 beta secretion in MDMs from patients with high plasma apolipoprotein B (ApoB), a common form of dyslipidemia (112). Surprisingly, LDL is not the only lipoprotein to exert Md-DAMP effects on human monocytes and macrophages. High-density lipoprotein (HDL) deficiency is associated with decreased circulating classical monocytes and increased non-classical monocytes in patients with metabolic syndrome (113).

Additionally, the *in vitro* culture of monocytes in the absence of HDL polarizes them towards the non-classical subgroup, characterized by a significant increase in IL-1β production (113). Concurring with this evidence, enrichment of serum medium with HDL abrogates macrophage differentiation towards the M1 proinflammatory profile, inhibiting the production of IL-6, TNF-alpha, MCP-1, and ROS (114). Interestingly, HDL prevents the inflammatory polarization of macrophages by redistributing caveolin-1 in the cell membrane and decreasing the phosphorylation of extracellular signal-regulated kinase (ERK) and signal transducer and activator of transcription 3 (STAT3) (114). As shown, lipoproteins such as oxLDL, native LDL, and HDL play crucial roles as Md-DAMPs, orchestrating the inflammatory activation of monocytes and macrophages and the production of cytokines that contribute to metainflammation and atherogenesis.

Lipids that participate in metainflammation are not limited to complex particles containing cholesterol, triglycerides, and apolipoproteins, but also SCFAs. SCFAs are single lipids produced by microbiota from dietary fiber that comprise a wide variety of fatty acids composed of less than six carbons, including 2-carbon acetic acid, 3-carbon propionic acid, 4-carbon butyric acid, 5-carbon valeric acid, and 6-carbon caproic acid (115). SCFAs serve as an energy source for various cell types, including colonocytes, hepatocytes, adipocytes, neurons, and immune cells. Besides energy metabolism, SCFAs exert Md-DAMP actions via G-protein-coupled receptors (GPRs) and modulation of TLR4-mediated signaling pathways, promoting either proinflammatory or anti-inflammatory responses depending on their concentration and target receptor (116). In this sense, 0.1 mM acetic acid decreases IL-6 and TNF-alpha production in the liver of mice with cecal ligation and puncture-induced sepsis, whereas 20 mM acetic acid stimulates IL-6, IL-8, and IL-1 beta synthesis in peripheral blood mononuclear cells (PBMCs) from healthy donors (117, 118). Propionic acid also exhibits bimodal effects on the inflammatory response. For instance, human intestinal epithelial tissue shows reduced levels of NFκB, MCP-1, and IL-8 when treated with low doses of propionic acid via MAPK activation. However, propionic acid concentrations above 20 mM induce the secretion of IL-6, IL-8, and IL-1 beta in human PBMCs via TLR4 (118, 119). Likewise, different butyric acid concentrations can elicit or suppress inflammatory pathways, as demonstrated in LPS-treated murine bone marrow-derived macrophages, which show decreased IL-6 production at 2 mM but no effect at 0.1 mM (120).

Interestingly, low SCFA thresholds appear to reflect a balance of gut microorganisms linked to immune tolerance. In contrast, an increase in SCFA production may indicate an imbalance in intestinal bacteria, leading to the inflammatory activation of innate immune cells. This idea concurs with prior data showing that butyrate concentrations below 0.5 mM promote the differentiation of naïve CD4+ T cells into IL-10-producing regulatory T cells via GPR41, suggesting that low SCFA thresholds can elicit immune tolerance (121). Conversely, supplementation with acetate, propionate, and butyrate at concentrations above 25 mM stimulates inflammatory activation of RAW264.7 macrophages, serving as a regulatory mechanism that elicits phagocytosis of *Klebsiella pneumoniae* and prevents dysbiosis (122). Another line of evidence supporting the duality of SCFA in promoting either a proinflammatory or an anti-inflammatory milieu is that SCFA concentration depends on the balance among gut microorganisms, including those belonging to the phyla Bacteroidetes, Actinobacteria, and Firmicutes (123). Therefore, dysbiosis may affect not only the balance of the gut microbiota but also SCFA production, favoring the development of inflammatory conditions or tolerogenic responses, a scenario that may lead to metainflammation and other related diseases such as T2D (124–127). Our understanding of SCFAs as Md-DAMPs is just emerging. However, the clear relationship among gut microbiota, SCFA concentration, and metainflammation reinforces the potential therapeutic use of SCFA supplementation to improve metabolic status, as shown in mice fed an HFD and in individuals with metabolic syndrome (128–130).

Uric acid is an organic heterocyclic compound containing carbon, nitrogen, oxygen, and hydrogen. Uric acid is also the end product of purine metabolism, which is filtered in the kidneys and excreted into the urine (131). A purine-enriched diet, including red meat, seafood, beer, and fructose-sweetened beverages, can increase uric acid production, leading to a condition called hyperuricemia when uric acid levels in the blood exceed 6 mg/dl in women or 7 mg/dl in men (132). Besides being a risk factor for developing gout and urolithiasis, uric acid is an Md-DAMP linking metainflammation with cardiovascular disease and metabolic dysfunction. A previous study from our research group demonstrated that uric acid stimulates the *in vitro* synthesis of TNF-alpha and TLR4 in human MDMs in a dose-dependent manner while also enhancing the phagocytic activity of these immune cells (76). Notably, the specific blockade of uric acid transport within macrophages abrogated both proinflammatory cytokine production and phagocytic activity, suggesting that uric acid exerts its effects by eliciting a receptor-dependent intracellular signaling cascade (76).

Previous evidence indicates that uric acid interacts with pattern recognition receptors (PRRs), including TLR2, TLR4, nucleotide-binding and oligomerization domain (NOD)-like receptors, and the nucleotide-binding domain, leucine-rich-containing family, pyrin domain-containing 3 (NLRP3) (133). Upon activation, TLR2 and TLR4 recruit MyD88 to interact with the L-1R-associated kinase (IRAK) protein family, leading to IκB phosphorylation and the release of NFκB (134). Along with promoting cytokine expression, NFκB activity aligns with ROS production, which serves as a triggering signal to activate NLRP3 and enhance macrophage inflammatory behavior in the presence of uric acid (135). This evidence supports previous reports that hyperuricemia enhances macrophage proinflammatory capacity and their ability to infiltrate atherosclerotic lesions in the aortas of ApoE-/- mice (136). Interestingly, inhibition of xanthine oxidase, the rate-limiting enzyme in purine degradation that produces uric acid, decreases IL-1 alpha and IL-1 beta gene expression in aortic lesions, also alleviating ROS production and plaque formation (137). Together, this body of experimental findings suggests that uric acid plays a role as an Md-DAMP, conferring inflammatory functions to macrophages through pathways involving urate transporters and NFκB and NLRP3-dependent signaling cascades.

Glucose is a monosaccharide, or simple sugar, that serves as the primary energy source for almost all living organisms (138). Conversely, excess glucose is a leading cause of morbidity and mortality worldwide due to the harmful effects of hyperglycemia on nerves and blood vessels that contribute to developing neuropathy, endothelial dysfunction, and vascular complications (139). Besides altering protein structure and function through glycation, weakening small blood vessels, and promoting ROS production, abnormally high blood glucose levels act as Md-DAMPs, triggering metainflammation and cardiometabolic dysfunction. Almost 10 years ago, our research team reported that 15 mM excess glucose, resembling the blood sugar amount in patients with uncontrolled T2D, promotes the *in vitro* polarization of human MDMs by increasing inducible nitric oxide synthase (iNOS) and decreasing IL-10 (140). A recent study expanded the body of evidence supporting the proinflammatory effects of glucose on human MDMs, revealing that hyperglycemia increases TLR4 expression, which partially explains the elevation of iNOS in these immune cells (141). High glucose can also increase IL-1 beta, IL-6, and TNF-alpha expression in human monocytes incubated with 24 mM glucose (142).

The molecular mechanisms by which excess glucose exerts its actions as an Md-DAMP remain uncertain but likely involve receptors such as TLR4 and the receptor for advanced glycation end-products (RAGE), glucose transporters such as GLUT1, and epigenetic mechanisms. In this sense, lactic acid and epigenetic changes are pivotal mediators of glucose’s actions. Briefly, accumulation of extracellular glucose leads monocytes and macrophages to increase glucose uptake via GLUT1, upregulating glycolysis that results in lactic acid overproduction (143). Lactate can also inhibit the prolyl hydroxylase domain (PHD), a dioxygenase family member that tags the transcription factor HIF-1α for proteasomal degradation (144). Lactate’s effect results in the accumulation of HIF-1α, promoting increased expression of cytokines and chemokines with inflammatory roles in monocytes and macrophages (145). High glucose prevents methylation of histone H3 (H3K9me3), a key inhibitor of proinflammatory gene expression (146). Sudden accumulation of intracellular glucose induces HIF-1α–dependent IL-6 expression, which activates transcription of the suppressor of variegation 3–9 homolog 1 (SUV39H1) in human and murine macrophages (147). SUV39H1 catalyzes the trimethylation of histone H3 at lysine 9 (H3K9me3), an epigenetic mechanism that acts as a negative feedback regulator of proinflammatory gene expression (148). Conversely, prolonged exposure to high intracellular glucose concentrations suppresses IL-6-mediated feedback, leading to repression of SUV39H1, inhibition of H3K9me3 methylation, and promotion of proinflammatory gene transcription (149). Collectively, this information highlights the profound effect of high glucose in triggering innate immune cell hyperactivity, which contributes to metainflammation that accompanies obesity, T2D, and cardiovascular disease. Understanding the molecular mechanisms triggered by Md-DAMPs, such as excess glucose, is crucial to explaining why monocytes and macrophages exhibit an inflammatory profile in hyperglycemia and how these cells contribute to the development of atherosclerosis.

**Figure 2:**
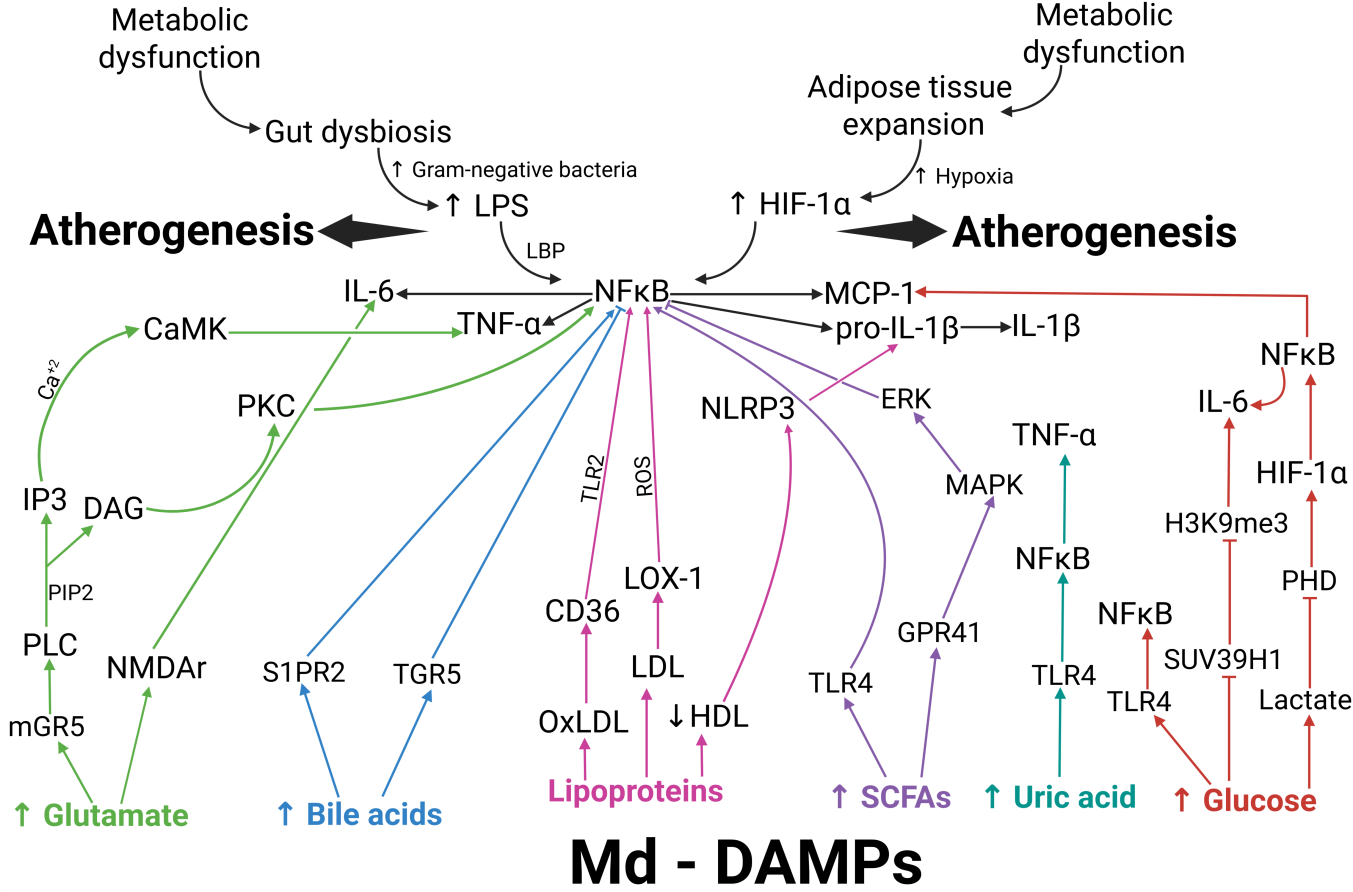
Map of the potential molecular interplay among entries of metainflammation, Md-DAMPs, target receptors, and intracellular mediators in atherogenesis. Metabolic dysfunction favors gut dysbiosis and adipose tissue expansion as primary entries of metainflammation. Gram-negative bacteria proliferate during gut dysbiosis, leading to increased LPS production. Once bound to LBP, LPS activates NFκB via TLR4. Adipose tissue expansion involves adipocyte hypertrophy and hyperplasia. Hypoxia increases as adipocytes expand, thereby activating HIF-1α. HIF-1α can also activate NFκB, thus promoting the expression of IL-6, TNF-alpha, pro-IL-1 beta, and MCP-1. Md-DAMPs are endogenous metabolic signals with the ability to stimulate monocytes, macrophages, and other immune cells to produce proinflammatory cytokines and chemokines via specific receptors and intracellular signaling pathways. After binding to mGR5 (green arrows), glutamate activates PLC, promoting PIP2 cleavage into IP3 and DAG. IP3 increases intracellular calcium, which leads to CaMK activation and TNF-alpha production. DAG activates PKC, which, in turn, leads to NFκB activation and TNF-alpha synthesis. Glutamate can also bind to NMDAr, which elicits IL-6 production. S1PR2 and TGR5 can recognize bile acids (blue arrows), thereby promoting NFκB activation and the expression of proinflammatory cytokines when bile acid levels increase. Ox-LDL acts through CD36 (pink arrows), thus inducing NFκB activation via TLR2. Native LDL exerts its effect via LOX-1 (pink arrows), which also activates NFκB through ROS production. Decreased HDL levels concur with NLRP3 activation, which cleaves pro-IL-1 beta to IL-1 beta (pink arrows). SCFAs exert their actions via TLR4 or GPR41 (purple arrows), promoting NFκB activation directly or via the ERK- and MAPK-dependent signaling pathways, respectively. Uric acid can bind to TLR4 (dark green), thereby activating NFκB and inducing TNF-alpha expression. Increased glucose acts through 3 different pathways (red arrows). After binding to TLR4, uric acid activates NFκB and TNF-alpha expression. Excess glucose inhibits SUV39H1 transcription, thereby suppressing H3K9me3-mediated methylation of the IL-6 gene. Increased intracellular glucose converts to lactic acid, which inhibits PHD activity and prevents proteasomal degradation of HIF-1α, thereby leading to NFκB activation. Metabolic dysfunction results in increased LPS production and HIF-1α activation, thereby triggering metainflammation that creates a milieu favorable to monocytes and macrophages for the production of proinflammatory cytokines and chemokines. In parallel, metabolic dysfunction also allows the release of Md-DAMPs, which directly stimulate monocytes and macrophages to synthesize proinflammatory cytokines and chemokines that can perpetuate and amplify metainflammation. The interplay between metainflammation igniters and Md-DAMPs favors atherosclerotic disease via specific receptors, including mGR5, NMDAr, S1PR2, TGR5, CD36, LOX-1, TLR4, and GPR41, as well as intracellular mediators such as PKC, NFκB, NLRP3, ERK, MAPK, and epigenetic mechanisms. An upward arrow indicates an increase. A downward arrow indicates a decrease. Connector arrows indicate a causative relationship, with the arrow pointing from the cause to the effect. Abbreviations: Md-DAMPs, metabolite-derived damage-associated molecular patterns; LPS, lipopolysaccharide; LBP, lipopolysaccharide-binding protein; HIF-1α, hypoxia-inducible factor 1 alpha; NFκB, nuclear factor kappa B; TNF-α, tumor necrosis factor alpha; IL-6, interleukin 6; pro-IL-1β, pro-interleukin 1 beta; IL-1β, interleukin 1 beta; PLC, phospholipase C; PIP2, phosphatidylinositol 4,5-bisphosphate; IP3, inositol trisphosphate; Ca^+2^, calcium; DAG, diacylglycerol; CaMK, calmodulin-dependent kinases; PKC, protein kinase C; S1PR2, sphingosine-1-phosphate receptor 2; TGR5, Takeda G-protein-coupled receptor 5; LDL, low-density lipoprotein; oxLDL, oxidized low-density lipoprotein; HDL, high-density lipoprotein; CD36, cluster of differentiations 36; TLR2, Toll-like receptor 2; LOX-1, lectin-like oxidized low-density lipoprotein receptor-1; ROS, reactive oxygen species; NLRP3, nucleotide-binding and oligomerization domain (NOD)-like receptors, and the nucleotide-binding domain, leucine-rich-containing family, pyrin domain-containing 3; SCFAs, short-chain fatty acids; TLR4, Toll-like receptor 4; GPR41, G-protein coupled receptor 41; ERK, extracellular-signal-regulated kinase; MAPK, mitogen-activated protein kinase; SUV39H1, suppressor of variegation 3–9 homolog 1; H3K9me3, trimethylation of histone H3 at lysine 9, PHD, prolyl hydroxylase domain.

As outlined, Md-DAMPs resulting from metabolic dysfunction can perpetuate metainflammation, initially driven by gut dysbiosis and adipose tissue expansion. Moreover, Md-DAMPs can stimulate immune cells to produce inflammatory factors and chemotactic agents that play prominent roles in atherosclerotic plaque formation, instability, and rupture (**Figure 2**).

## Integrating the molecular mechanisms of metainflammation in atherosclerosis

**Figure 3:**
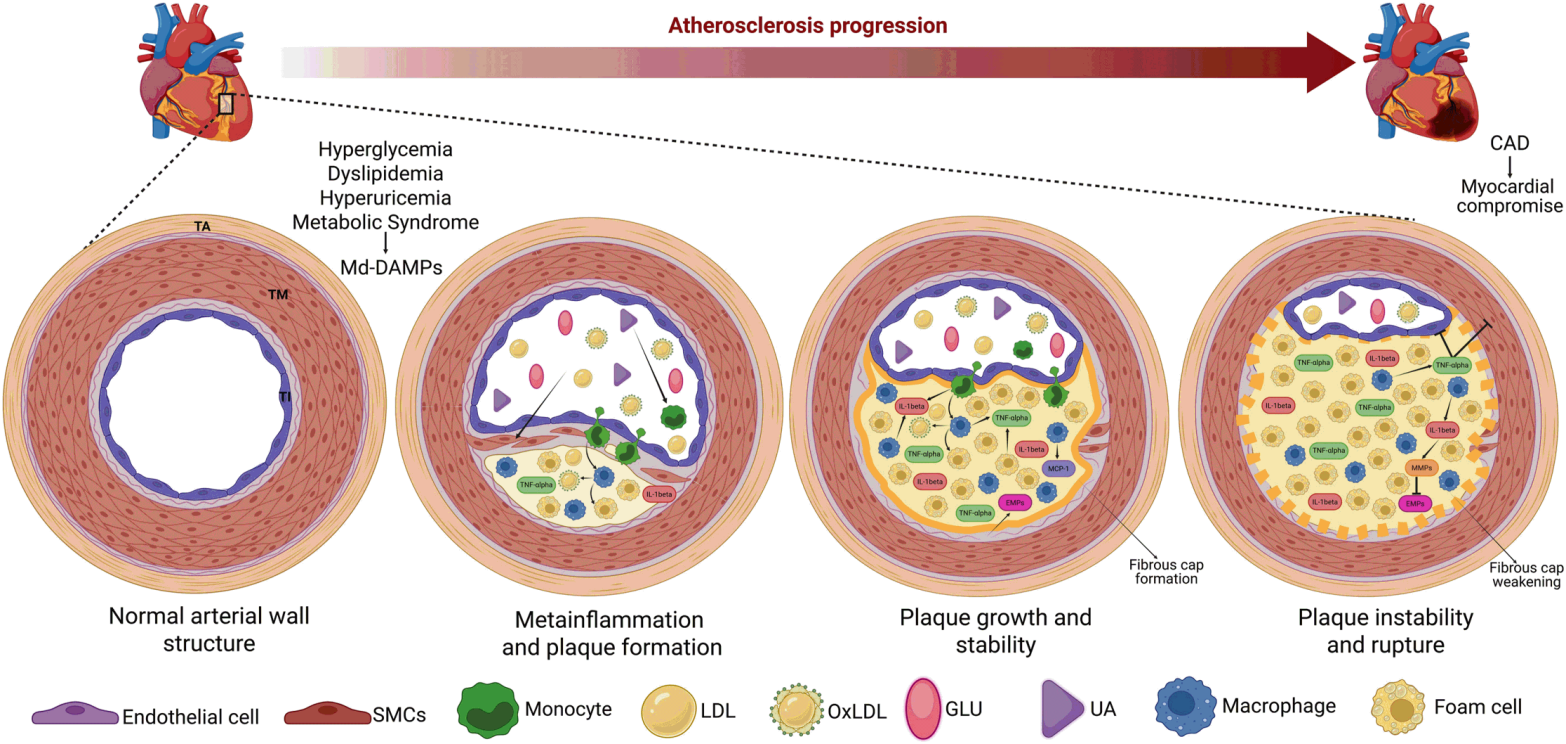
Gut dysbiosis and adipose tissue expansion are crucial triggers of TNF-alpha production and metainflammation. The abnormal increases in cholesterol, FFAs, LDL, glucose, and uric acid, among others, contribute to the development of dyslipidemia, hyperuricemia, hyperglycemia, and metabolic syndrome. These metabolic entities also serve as a source of Md-DAMPs, thereby perpetuating the abnormal increase in these immune triggers in circulation. Md-DAMPs provide micronutrients that alter the balance of microorganism species composing the gut microbiome, resulting in intestinal dysbiosis. Intestinal dysbiosis promotes the proliferation of gram-negative bacteria capable of producing LPS. LPS can cross the intestinal barrier and reach systemic circulation upon binding to LBP, thereby stimulating monocytes and tissue-resident macrophages to produce TNF-alpha and other inflammatory mediators that can trigger metainflammation. In parallel but not necessarily dependent, Md-DAMPs can also directly reach adipocytes within the adipose tissue, resulting in tissue hyperplasia and hypertrophy. The process of adipose tissue expansion induces hypoxia, primarily affecting adipocytes distant from the vascular bed, thereby increasing HIF-1α expression. HIF-1α is a master transcription factor that stimulates adipocytes, monocytes, and macrophages to synthesize TNF-alpha via NFκB, thereby favoring the development of metainflammation. As outlined, gut dysbiosis and adipose tissue expansion converge on TNF-alpha production, a cytokine with potent proinflammatory actions that also triggers metainflammation, immune cell hyperactivity, and metabolic dysfunction. An upward arrow indicates an increase. Bold connector arrows indicate a causative relationship, with the arrow pointing from the cause to the effect. Abbreviations: FFAs, free-fatty acids; LDL, low-density lipoprotein; Md-DAMPs, metabolite-derived damage-associated molecular patterns; LPS, lipopolysaccharide; LBP, lipopolysaccharide-binding protein; TNF-alpha, tumor necrosis factor alpha; HIF-1α, hypoxia-inducible factor 1 alpha; NFκB, nuclear factor kappa B.

We have reviewed the pathophysiological mechanisms by which gut dysbiosis-derived LPS and adipose tissue expansion trigger metainflammation, with particular emphasis on the activity of monocyte subsets and macrophages. We also elucidated the molecular basis of Md-DAMP function in preserving and amplifying receptor-mediated intracellular signaling pathways that lead to immune cell hyperactivation, proinflammatory cytokine production, and ROS release. Now, we will outline how these actors interact with each other in atherosclerotic plaque formation and rupture, causing acute heart attacks and strokes (**Figure 3**).

Atherosclerotic plaque formation primarily occurs in arteries, a vast component of the circulatory system consisting of muscular and elastic vessels that allow transport of oxygen, nutrients, and hormones from the heart to the rest of the body. Arteries are composed of three superimposed tissue layers, named the tunica intima, the tunica media, and the tunica adventitia. The tunica intima is the innermost layer of the artery, primarily composed of a flattened endothelial cell lamina supported by collagen-enriched elastic fibers. The tunica media plays a critical role in regulating vessel tone due to the presence of smooth muscle cells superimposed around the tunica intima. The tunica adventitia is the artery’s outermost layer, mainly consisting of collagen and elastic fibers, fibroblasts, autonomic nervous fibers, and the vasa vasorum, which together regulate the vessel tone and the attachment of the artery to surrounding tissues.

Atherosclerotic plaque formation occurs in the tunica intima through a complex process in which Md-DAMPs, monocytes, macrophages, and other inflammatory immune cells interact to promote endothelial damage and dysfunction in a first stage. As mentioned, metabolic alterations serve as the primary source of Md-DAMPs, including glutamate, bile acids, SCFAs, uric acid, and glucose, which can promote damage to the inner lining of arteries and lead to endothelial dysfunction. In this regard, prior studies have demonstrated that endothelial cells express both NMDAr and mGR5 (150). In the presence of high extracellular glutamate thresholds, NMDAr can activate Ca^2+^-permeable channels in endothelial cells, increasing intracellular Ca^2+^ and potentially inducing mitochondrial Ca^2+^ overload, ROS generation, vasoconstriction, and endothelial injury (151). In parallel, glutamate interaction with mGR5 on endothelial cells activates the PLC-dependent PKC signaling pathway, leading to NFκB activation and production of TNF-alpha and MCP-1, which mediate immune cell infiltration in the tunica intima (152). Furthermore, bile acid-induced S1PR2 activation plays a critical role in triggering the NFκB-dependent signaling pathway that mediates proinflammatory cytokine release, ROS production, and vascular endothelial damage (153). Similarly, butyrate levels above 5 mM can stimulate the PKC, ERK, and MAPK-dependent signaling pathways via GPR41, resulting in activation of NFκB and AP-1, and production of TNF-alpha and adhesion molecules that cooperatively enhance leukocyte transmigration and vascular inflammation (154). Elevated uric acid levels also induce endothelial dysfunction by stimulating NADPH oxidase activity, leading to superoxide anion production and peroxynitrite formation, which reduces nitric oxide bioavailability and vasodilation (155). Excess glucose is another Md-DAMP that promotes endothelial dysfunction through impairing nitric oxide bioavailability and vascular tone, and increasing oxidative stress, immune cell infiltration, and tissue damage via TLR4, RAGE, and GLUT1 (156). As shown, Md-DAMP effects on the vascular endothelium involve numerous receptors and signaling pathways that result in the release of proinflammatory cytokines, ROS production, immune cell adhesion and transmigration, impaired vascular tone, and endothelial injury.

In turn, endothelial injury produces multiple DAMPs, including heat shock proteins (HSPs), high-mobility group box 1 (HMGB1), and mitochondrial deoxyribonucleic acid (mtDNA) (157, 158). Together with Md-DAMPs, these DAMPs can recruit circulating monocytes and other immune cells to the vascular endothelium. Monocytes infiltrating the tunica intima can differentiate into macrophages and adopt an inflammatory activation profile instigated not only by DAMPs but also by Md-DAMPs, which can extend cytokine production and act as signals to recruit additional innate cells (159). This microenvironment influences macrophages to engulf cholesterol, triglycerides, lipoproteins, and oxLDL accumulated in the injured endothelium, converting macrophages into foam cells (160). These foam cells not only contribute to plaque buildup but also exacerbate metainflammation by producing MCP-1, CXC-chemokine ligand 1 (CXCL1), and CXCL4, which recruit additional leukocytes and platelets and promote monocyte arrest in the atheroma (161, 162). Furthermore, foam cells within the atherosclerotic plaque can synthesize proinflammatory cytokines that perpetuate the inflammatory milieu, including IL-1 beta, IL-6, and TNF-alpha (163). In the absence of changes in habits or proper pharmacotherapy, the atheroma continues to grow due to the accumulation of fats, cell debris, calcium, platelets, monocytes, macrophages, and foam cells, leading to narrowing and hardening of the arteries, also referred to as atherosclerosis. As seen, atheroma formation is the first stage of atherosclerotic disease.

Afterward, the body must stabilize the plaque to prevent it from entering the bloodstream and produce a fibrous cork composed mainly of collagen and extracellular matrix proteins (164). However, the atherosclerotic plaque continues to secrete a variety of cytokines and DAMPs, which affect its stability and can lead to weakening and rupture. In this sense, TNF-alpha can mediate not only inflammatory responses but also increase plaque instability by inducing apoptosis of smooth muscle cells and endothelial cells within the vascular endothelium (165). Previous evidence shows that TNF-alpha stimulates caspase-3 activity in human vascular smooth muscle cells cultured *in vitro*, which may explain the reduced proliferation and increased apoptosis observed in these cells (166). A recent study reported that human umbilical vascular endothelial cells (HUVECs) exhibit caspase-3-mediated apoptosis when treated with TNF-alpha (167). At the same time, another study found that the apoptosis rate in HUVECs increased with increasing TNF-alpha dose (168). Besides playing a role in inflammasome activation, IL-1 beta is particularly active in atherogenesis due to its ability to promote the expression of matrix metalloproteinases (MMPs), which are key contributors to fibrous cap degradation and plaque instability (169). MMP-1 can degrade type I and III collagen, while MMP-2 and MMP-9 can cleave type IV collagen in both animal models and *in vitro* cell cultures, supporting their roles in plaque instability and rupture (170, 171).

Md-DAMPs not only participate in atherosclerotic plaque rupture by orchestrating TNF-alpha and IL-1 beta expression but also by inducing apoptosis of endothelial cells, a crucial element of arteries. For instance, 30 mM glucose activates NFκB, upregulating cyclooxygenase-2 (COX-2) and enabling caspase-3 activation, thereby increasing apoptosis in HUVECs (172). Later, another research team replicated these findings in human coronary artery endothelial cells, confirming that excess glucose can weaken atherosclerotic plaque (173). Consistent with increased apoptosis in HUVECs, uric acid also promotes the expression of caspase-3 and B-cell lymphoma 2 (Bcl-2) in a time- and dose-dependent manner (174). Furthermore, recent information revealed that 4 mM of butyric acid provokes DNA damage-related cell apoptosis in HUVECs, thus corroborating emerging evidence linking gut dysbiosis to atherosclerotic disease (175, 176). As outlined, this emerging information consistently shows that proinflammatory cytokines and Md-DAMPs can contribute to weakening atherosclerotic plaque by affecting the viability of smooth muscle cells and endothelial cells, as well as diminishing extracellular matrix protein content. This scenario elicits fibrous cap rupture and thrombus formation, blocking the blood flow within arteries and leading to acute myocardial infarction and cerebrovascular accident (**Figure 3**).

## Conclusion

We have examined the complex interplay between gut dysbiosis-derived LPS and adipose tissue expansion as crucial signals that unleash low-grade systemic inflammation during metabolic dysfunction, also known as metainflammation. This state of metainflammation enables the release of Md-DAMPs as glutamate, bile acids, lipoproteins, butyrate, uric acid, and excess glucose, which can perpetuate proinflammatory polarization of monocytes and macrophages. Md-DAMPs predispose circulating monocytes toward proinflammatory activity by increasing the CD14+CD16+ non-classical monocyte subset, IL-1β synthesis, and monocyte infiltration into inflamed tissues, such as visceral adipose tissue. Priming of monocytes toward an inflammatory profile predisposes macrophages to classical activation once these immune cells have differentiated in peripheral tissues, thereby perpetuating the release of cytokines with proinflammatory actions, such as TNF-alpha. Md-DAMPs exert their inflammatory capacity through specific receptors and intracellular signaling pathways, including mGR1, mGR5, NMDAr, FXR, PXR, VDR, S1PR2, TGR5, CD36, LOX-1, TLR4, GPR41, urate transporter 1 (URAT1), GLUT1, RAGE, PLC, PKC, ERK, and MAPK. This variety of receptors with metabolic functions can also activate molecular mediators such as HIF-1α, CaMK, NFκB, AP-1, NFAT, and NLRP3, which in turn induce the expression of TNF-alpha, IL-1 beta, IL-6, and MCP-1, among others. The role of Md-DAMPs in the humoral and cellular responses associated with metainflammation becomes particularly relevant in atherogenesis, where monocytes, macrophages, and foam cells actively contribute to the formation and rupture of atherosclerotic plaques. Md-DAMPs such as glucose, uric acid, or butyrate continue to stimulate macrophages and foam cells to produce TNF-alpha and IL-1 beta within the atheroma, leading to remodeling of the vascular endothelium. The mechanisms by which Md-DAMPs and proinflammatory cytokines contribute to atherosclerotic plaque instability involve the apoptosis of smooth muscle cells and endothelial cells within the atheroma, as well as the degradation of extracellular matrix proteins. Ultimately, the intricate interplay among Md-DAMPs, monocytes, macrophages, foam cells, and cytokines culminates in atherosclerotic plaque rupture and thrombus formation, both of which are key pathophysiological features of cardiovascular disease. The current knowledge presented in this comprehensive review may help elucidate the molecular basis behind the role of gut dysbiosis-derived LPS and adipose tissue expansion in initiating metainflammation. Moreover, the experimental and clinical evidence discussed here may contribute to a better understanding of the molecular mechanisms through which Md-DAMPs can perpetuate metainflammation by increasing the activity of cytokine-producing immune cells. Collectively, this information may help refresh our conceptual understanding of atherogenesis to identify new molecular targets against immune cell receptors and signaling pathways involved in the development of metainflammation and atherosclerosis.
